# miR-26a Suppresses Tumor Growth and Metastasis by Targeting FGF9 in Gastric Cancer

**DOI:** 10.1371/journal.pone.0072662

**Published:** 2013-08-28

**Authors:** Min Deng, Hai-lin Tang, Xi-hong Lu, Mei-yuan Liu, Xiao-min Lu, Yi-xue Gu, Ji-fang Liu, Zhi-min He

**Affiliations:** 1 Cancer Hospital and Cancer Research Institute, Guangzhou Medical University, Guangzhou, China; 2 Department of Breast Oncology, Sun Yat-Sen University Cancer Center, Guangzhou, China; 3 Cancer Research Institute, University of South China, Hengyang, China; 4 Affiliated Nanhua Hospital, University of South China, Hengyang, China; Institute of Molecular Medicine, Taiwan

## Abstract

The role of miR-26a in cancer cells seemed controversial in previous studies. Until now, the role of miR-26a in gastric cancer remains undefined. In this study, we found that miR-26a was strongly downregulated in gastric cancer (GC) tissues and cell lines, and its expression levels were associated with lymph node metastasis and clinical stage, as well as overall survival and replase-free survival of GC. We also found that ectopic expression of miR-26a inhibited GC cell proliferation and GC metastasis *in vitro* and *in vivo*. We further identified a novel mechanism of miR-26a to suppress GC growth and metastasis. FGF9 was proved to be a direct target of miR-26a, using luciferase assay and western blot. FGF9 overexpression in miR-26a-expressing cells could rescue invasion and growth defects of miR-26a. In addition, miR-26a expression inversely correlated with FGF9 protein levels in GC. Taken together, our data suggest that miR-26a functions as a tumor suppressor in GC development and progression, and holds promise as a prognostic biomarker and potential therapeutic target for GC.

## Introduction

miRNAs are endogenously expressed, small noncoding RNAs that negatively regulate gene expression by causing degradation of target mRNAs, inhibition of the translation of these mRNAs or both [1]. miRNAs take a part in crucial cellular processes such as the stress response, development, differentiation, apoptosis, and proliferation [2]. Altered miRNA expression has been reported in numerous malignancies, including breast [3], [4], lung [5], liver [6], stomach [7], colon [8], brain [9], leukaemia [10], and lymphoma [11]. An increasing number of studies have demonstrated that miRNAs can function as oncogenes or tumor suppressors, and they are often dysregulated in tumors [12], [13]. In this regard, oncogenic miRNAs are frequently upregulated, whereas tumor-suppressing miRNAs are downregulated in tumors. For instance, let-7 has been reported to be underexpressed in lung cancer and to target the oncogenic Ras [14], [15]. We previously reported that miR-216b is strongly downregulated in nasopharyngeal carcinoma and attenuates tumour growth by targeting KRAS [16]. In contrast, the oncogenic miR-17-92 cluster of miRNAs are upregulated in variety of tumor [17], and enforced expression of these miRNAs in the well-studied Eμ-myc transgenic mouse model of B cell lymphoma dramatically accelerates disease onset and progression [18]. However, the role of miR-26a in cancer cells seemed controversial, as it is a tumor suppressor in hepatocellular carcinoma [19], breast cancer [20] and nasopharyngeal carcinoma [21], [22] but is an oncogene in glioma [23] and cholangiocarcinoma [24]. Until now, the role of miR-26 in gastric cancer was undefined.

In the present study, we examined miR-26a expression in 40 paired normal and GC specimens by quantitative RT-PCR (qRT-PCR) analysis. miR-26a was found to be strongly downregulated in GC tissues as compared with that of adjacent normal stomach tissues. This result was further confirmed by in situ hybridization on tissue microarrays consisting of 126 cases of GC tissues and 41 cases of adjacent normal tissues. Moreover, decreased miR-26a was associated with poor prognosis and might independently predict overall survival (OS) and replase-free survival (RFS) in gastric cancer. Functional studies revealed that miR-26a suppressed GC cell growth and metastasis by targeting FGF9.

## Results

### miR-26a is Downregulated in Human Gastric Cancer

**Figure 1:**
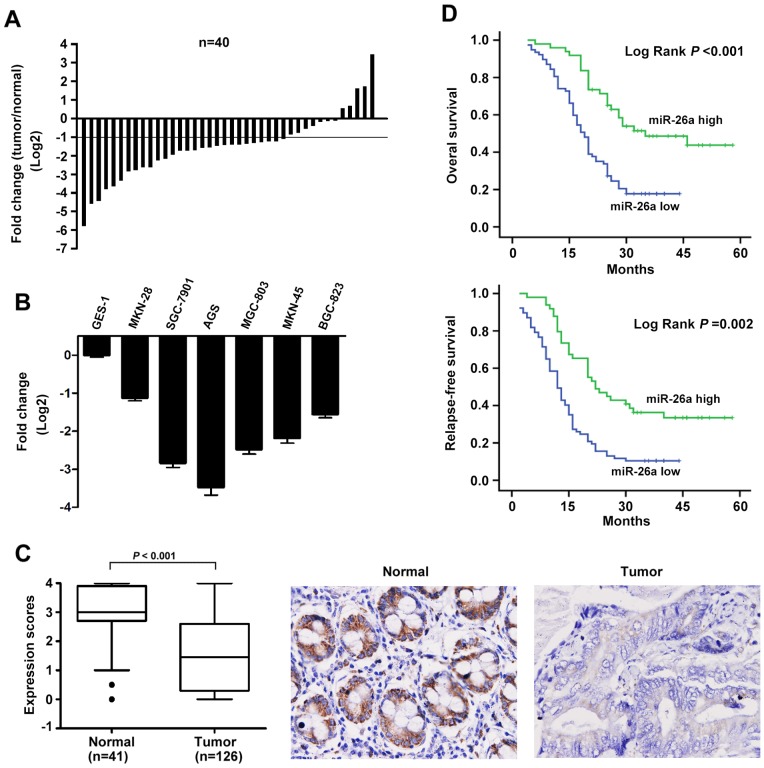
miR-26a is frequently downregulated in human gastric cancer and correlates with GC prognosis. (A) miR-26a was detected in 40 gastric cancer patients by qRT-PCR. Data are shown as log2 of the fold change in gastric cancer tissues (tumor) relative to adjacent normal tissues (normal). (B) Relative expression of miR-26a in 6 cell lines derived from gastric cancer and one nonmalignant gastric cell line (GES-1) was determined by qRT-PCR. Data are shown as log2 of the fold change in GC cell lines relative to GES-1. (C) miR-26a expression was analyzed in adjacent normal tissues (Normal) and GCs (Tumor) samples on the tissue microarrays by in situ hybridisation. Expression scores are shown as box plots. Representative images of miR-26a expression by in situ hybridization are shown. Original magnification: ×200. (D) Survival analysis of GC. OS and RFS curves for 126 GC patients with high or low miR-26a expression were constructed using the Kaplan-Meier method and evaluated using the log-rank test.

Using a qRT-PCR method, miR-26a levels were detected in 40 pairs of gastric cancer tissues and their matched adjacent tissues, as well as gastric cell lines. Among the 40 patients with gastric cancer, approximately 70% (28 of 40 patients) of tumors revealed a more than two-fold reduction in miR-26a levels, with a 5.76-fold reduction relative to adjacent normal tissues, suggesting that reduction of miR-26a was a frequent event in human GC (Figure 1A). Moreover, miR-26a expression was reduced in all gastric cancer cell lines (MKN-28, SGC-7901, AGS, MGC-803, MKN-45, and BGC-823) compared with the nonmalignant gastric cell line GES-1 (Figure 1B). To further verify the results concerning the biological role of miR-26a in human gastric carcinogenesis, we employed in situ hybridisation to evaluate miR-26a expression in 126 GCs and 41 non-tumor tissues on tissue microarrays (TMAs). The results revealed that the expression scores of miR-26a were significantly decreased in GCs compared with normal tissues (Figure 1C). Next, we determined the potential clinicopathological implications of altered miR-26a expression. Clinical samples were divided into low expression and high expression groups based on miR-26a expression scores greater or less than the median. Consistent with the above data, out of 41 total normal stomach samples, 35 (85%) had high expression of miR-26a. In contrast, 60% (76 of 126) of gastric carcinoma specimens had low to negative expression of miR-26a. Of the 126 individuals with GC, 38 were negative for lymph node metastasis, and 45% of these patients had low to negative expression of miR-26a (Table 1). Eighty-eight patients were positive for lymph node metastasis, and 67% of them showed downregulation or loss of miR-26a (Table 1). Moreover, we found low expression of miR-26a in 37% and 77% of gastric tumors classified as stage I/II and stage III/IV, respectively (Table 1). Therefore, miR-26a expression inversely correlates with lymph node metastasis and clinical stage (*P* = 0.019 and *P*<0.001, respectively). However, miR-26a did not correlate with age, gender, cell differentiation, or invasion depth (T stage). These results suggest that the miR-26a might play a critical role in the GC metastasis and progression.

**Table 1:** Analysis of the correlation between the expression of miR-26a in primary GC and its clinicopathological parameters.

Viable	Cases	miR-26a		
		low	high	P value
Age (years)				
<60	73	42	31	0.454
≥60	53	34	19	
Gender				
Male	70	43	27	0.776
Female	56	33	23	
Histological grade^a^				
Well and Moderate	32	21	11	0.477
Poor & Other	94	55	39	
T stage				
T1–T2	71	39	32	0.106
T3–T4	55	38	17	
TNM stage				
I–II	51	19	32	<0.001
III–IV	75	58	17	
Lymph node metastasis				
Present	88	59	29	0.019
Absent	38	17	21	

a Well-differentiated adenocarcinoma (Well), moderately differentiated adenocarcinoma (Moderate), poorly differentiated adenocarcinoma (Poor), Other histological type (Other).

### Decreased miR-26a Correlates with Poor Clinical Prognosis

To further analyze the significance of miR-26a in terms of clinical prognosis, Kaplan-Meier survival analysis was performed using patient overall survival and relapse-free survival. The results demonstrated that patients with low miR-26a expression had shorter median OS and RFS than did patients with high miR-26a expression (21.6 months vs. 39.0 months, *P*<0.001 for OS; 15.2 months vs. 31.6 months, *P* = 0.002 for RFS; Figure 1D). We used Cox proportional-hazards regression to further evaluate the association between miR-26a expression and prognosis. In univariate analysis, lymph node metastasis, TNM stage, and miR-26a levels were significantly associated with the OS and RFS (Table 2, 3). The final multivariate model revealed that overall survival time significantly depended on lymph node metastasis, TNM stage, and miR-26a levels (Table 2), and relapse-free survival time did on lymph node metastasis and miR-26a levels (Table 3).

**Table 2:** Univariate and multivariable Cox regression analysis of overall survival (Cox proportional hazards regression model).

Viable	Univariate analysis			Multivariate analysis		
	RR	95% CI	P value	RR	95% CI	P value
Age (<60 vs. ≥60)	0.82	0.52–1.29	0.387	–	–	–
Gender (male vs. female)	0.96	0.62–1.48	0.860	–	–	–
Histological grade (well andmoderate vs. poor and other)	1.15	0.71–1.87	0.568	–	–	–
T stage (T1–T2 vs. T3–T4)	0.97	0.55–1.71	0.915	–	–	–
TNM stage (I–II vs. III–IV)	2.12	1.00–4.49	0.050	1.87	1.14–3.05	0.012
Lymph node metastasis(absent vs. present)	1.92	1.04–3.55	0.038	2.03	1.09–3.78	0.025
miR-26a (low vs. high)	0.57	0.34–0.97	0.040	0.59	0.35–0.98	0.040

Abbreviations: RR, relative risk; CI, confidence interval.

**Table 3:** Univariate and multivariable Cox regression analysis of relapse-free survival (Cox proportional hazards regression model).

Viable	Univariate analysis			Multivariate analysis		
	RR	95%CI	P value	RR	95%CI	P value
**Univariate analysis**						
Age (<60 vs. ≥60)	0.79	0.52–1.21	0.280	–	–	–
Gender (male vs. female)	0.93	0.62–1.39	0.732	–	–	–
Histologic grade (well andmoderate vs. poor and other)	1.12	0.72–1.76	0.613	–	–	–
T stage (T1–T2 vs. T3–T4)	0.79	0.45–1.40	0.421	–	–	–
TNM stage (I–II vs. III–IV)	1. 90	0.94–3.89	0.075^a^	1.57	0.95–2.60	0.076^a^
Lymph node metastasis(absent vs. present)	2.52	1.43–4.45	0.001	2.53	1.47–4.35	0.001
miR-26a (low vs. high)	0.59	0.37–0.95	0.031	0.59	0.37–0.92	0.022

a *P*<0.1.

### miR-26a Suppresses GC Growth and Metastasis

**Figure 2:**
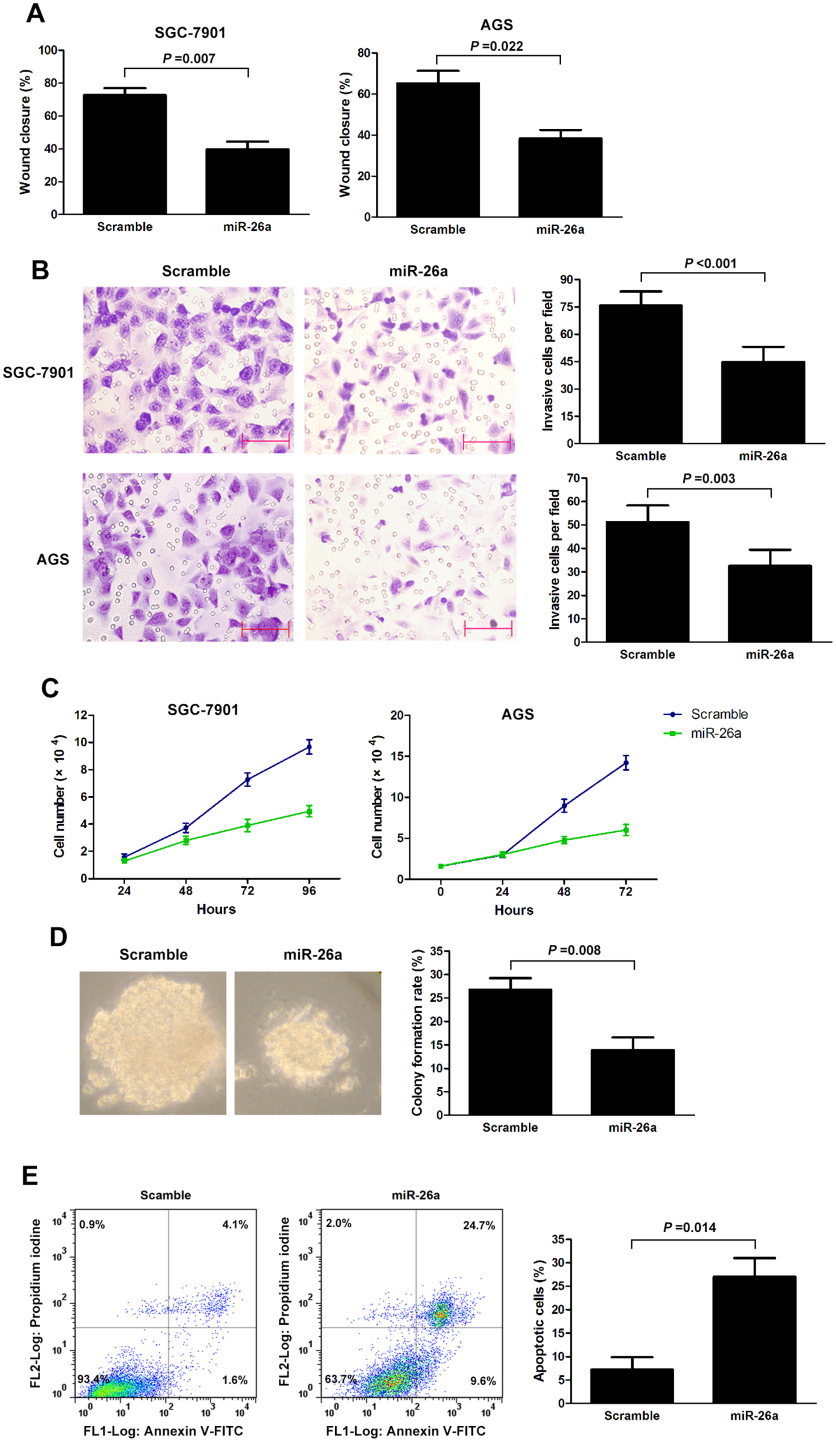
miR-26a inhibits cell migration, invasion, growth and soft agar colony formation, and induces cell apoptosis in GC. (A, B) The wound healing assay (A) and invasion assay (B) of SGC-7901 and AGS cells infected with miR-26a or scramble lentivirus. The invasion assay was measured by way of Transwell assays with Matrigel. (C) The growth of SGC-7901 and AGS cells infected with miR-26a or scramble lentivirus was assayed. (D) Colony growth assays in soft agar were performed on SGC-7901 with overexpression of miR-26a or scramble. Representative images of the assays are shown (left panel). Original magnification: ×200. (E) Overexpression of miR-26a induces tumor cell apoptosis. SGC-7901 cells were infected with miR-26a or scramble lentivirus. The apoptotic cells were evaluated by Annexin V-FITC and propidium iodine staining and analyzed with FACS. All data are presented.as mean ± s.e.m from at least three separate experiments.

Noting the inverse correlation between miR-26a levels and metastasis, we investigated the effect of miR-26a re-expression on the migration and invasion abilities of GC cell lines. Two GC cell lines (SGC-7901 and AGS) with relatively low basal expression of miR-26a (Figure 1B) were infected with either miR-26a or control lentivirus and selected with 5 mg/l puromycin for two weeks. Next, wound healing assay and transwell assay were performed. As expected, overexpression of miR-26a significantly suppressed cell migration and invasion abilities (Figure 2A, B; Figure S1).

To demonstrate the effect of miR-26a on GC growth, we performed GC cell proliferation assay. The proliferation assay showed that ectopic expression of miR-26a in SGC-7901 and AGS attenuated cell proliferation compared with control cells (Figure 2C). Moreover, ectopic miR-26a expression inhibited colony formation ability in soft agar (Figure 2D). We then performed cell apoptosis analysis and revealed that miR-26a overexpression in SGC-7901 induced cell apoptosis (Figure 2E).

**Figure 3:**
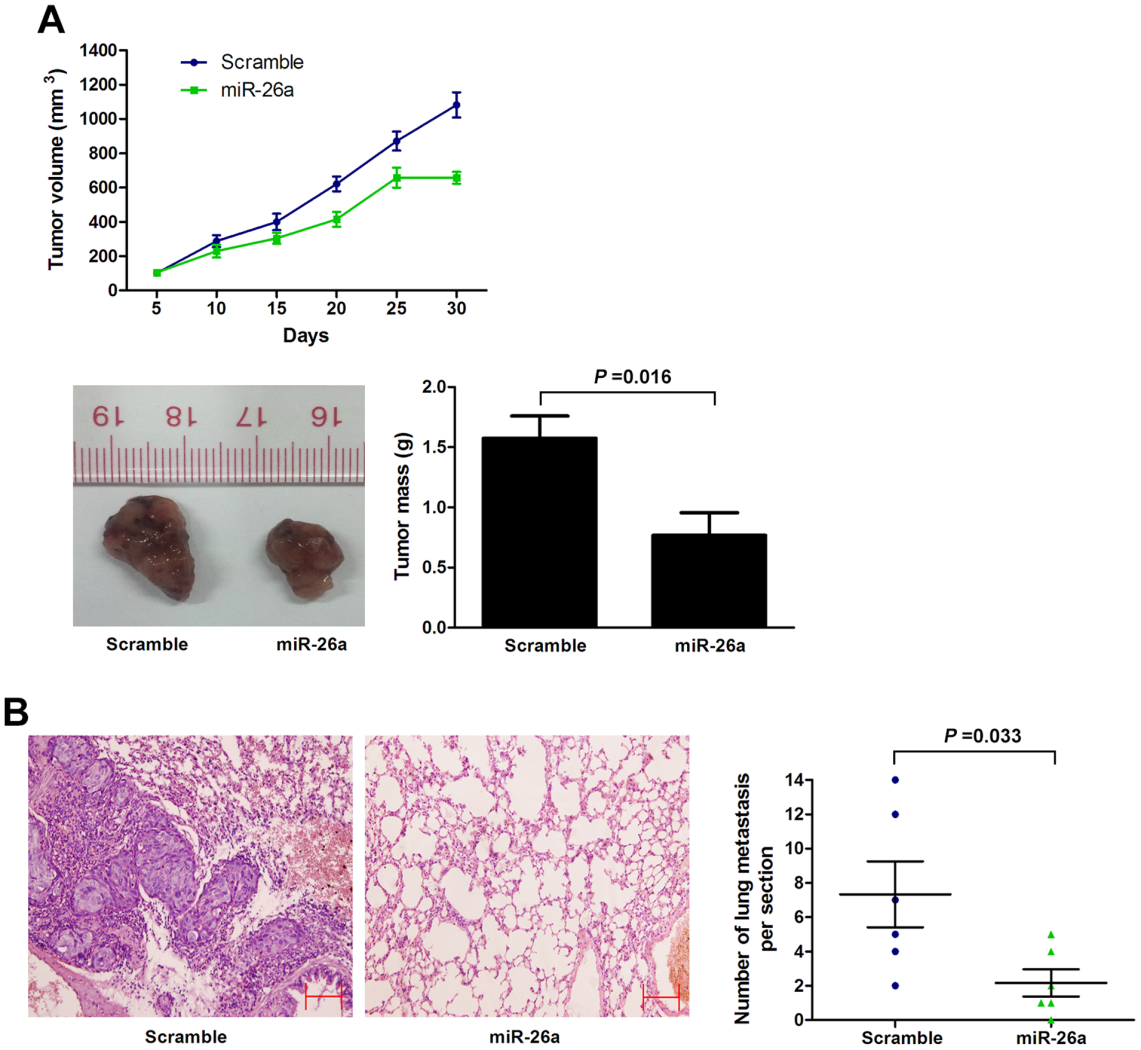
miR-26a inhibits GC cell growth and metastasis in vivo. (A) Tumor growth in mouse xenograft models. SGC-7901 cells infected with miR-26a or scramble lentivirus were injected subcutaneously into nude mice. Tumor size was measured every 5 days. After 30 days, the mice were killed, necropsies were performed, and tumors were weighed. (B) Tumor metastasis in mouse xenograft models. SGC-7901 cells with overexpression of miR-26a or scramble were injected into the tail vein of nude mice. After 45 days, the mice were killed. micrometastases in lung per HE-stained section in individual mice were calculated. Each group had six mice. Original magnification, ×100; scale bar: 50 µm. All data are shown as mean±s.e.m.

Next, we tested whether miR-26a could play a role in tumorigenesis by using nude mouse xenograft models. We found that overexpression of miR-26a in SGC-7901 cells significantly suppressed tumor growth in nude mice (Figure 3A; Figure S2). Then, SGC-7901 cells infected with either miR-26a or control lentivirsus were injected into the tail vein of nude mice to examine lung metastasis. As shown Figure 3B, a significantly lower number of macroscopic lung metastases could be observed in miR-26a overexpressing cells than control cells. These results indicate that miR-26a may repress GC growth and metastasis.

### miR-26a Directly Targets and Inhibits FGF9

**Figure 4:**
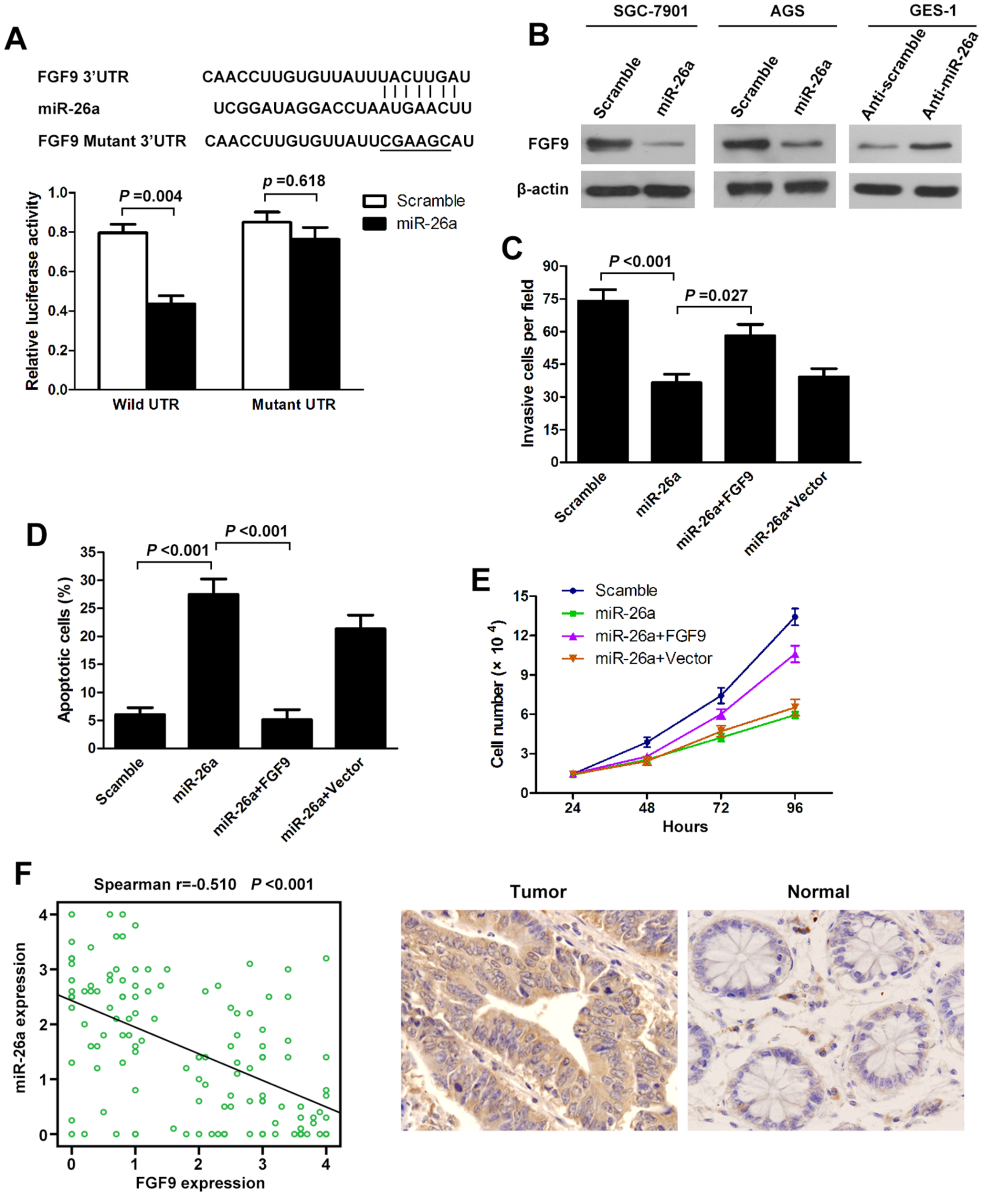
miR-26a directly targets FGF9. (A) The 3′-UTR element of FGF9 messenger RNA is partially complementary to miR-26a. miR-26a or scramble control and luciferase reporter containing either a wildtype or a mutant 3′-UTR were co-transfected into HEK-293T cells. And a Renilla luciferase expressing construct exerts as internal control. (B) Western blot analysis of FGF9 expression in SGC-7901 and AGS cells infected with miR-26a, and GES-1 transfected with miR-26a inhibitors (Anti-miR-26a). (C, D, E) FGF9 abrogates the suppressive roles of miR-26a in GC cell invasion and growth. SGC-7901 cells stably expressing miR-26a or scramble were transfect with or without FGF9 plasmids. Invasion assays(C), Apoptosis analysis (D), and Cell proliferation analysis (E) were performed with the above cells as described in Materials and Methods. Data are presented as mean±s.e.m from at least three independent experiments. (F) Spearman’s correlation scatter plot of the levels of miR-26a (determined by in situ hybridization) and FGF9 protein (determined by immunohistochemistry) in 126 GC specimens. Representative images of FGF9 expression by immunohistochemistry are shown (right panel). Original magnification: ×200.

To understand how miR-26a suppresses GC growth and metastasis, we used three algorithms (Targetscan, Pictar and Miranda) to help identify miR-26a targets in human gastric cancers. Of these target genes that were predicted by all three algorithms (Table S1), FGF9 attracted our attention immediately as it has been implicated in tumorigenesis or metastasis [26]–[28]. We cloned the full-length FGF9 3′-UTR into a luciferase reporter vector. Luciferase assay revealed that miR-26a directly bound to FGF9 3′-UTR, and by which it remarkably reduced luciferase activities (Figure 4A). However, mutation of the putative miR-26a sites in the 3′-UTR of FGF9 abrogated luciferase responsiveness to miR-26a (Figure 4A). To directly assess the effect of miR-26a on FGF9 expression, we performed western blot analysis. As seen in Figure 4B, lentiviral induced ectopic miR-26a dramatically suppressed the FGF9 protein levels in SCG-7901 and AGS cells. Furthermore, knockdown of miR-26a, through transfection of anti-miR-26a, in GES-1 cells increased FGF9 protein levels (Figure 4B; Figure S1). Taken together, these results indicate that FGF9 is a direct downstream target for miR-26a in GC cells. The above results prompted us to examine whether miR-26a suppresses GC growth and metastasis through repressing FGF9 expression. For this purpose, FGF9 was re-expressed in miR-26a-transfected SGC-7901 cells. In miR-26a-expressing cells, re-expression of FGF9 rescued the invasion and growth defects of miR-26a (Figure 4C, D, E). Finally, we tested if miR-26a expression correlated with FGF9 protein levels in GC. There was an inverse correlation between the FGF9 protein levels, indicated by immunohistochemistry staining, and miR-26a expression assessed by in situ hybridization in 126 GC tissues on TMAs as used above (Figure 4F; Figure1C). Our findings demonstrate that miR-26a has properties consistent with tumor suppressor function. The ability to modulate FGF9 levels might explain, at least in part, why miR-26a can inhibit GC growth and metastasis.

## Discussion

miR-26a belongs to the miR-26 family, which contains another member miR-26b, both of which house identical sequence with the exception of 2 different nucleotides in mature miRNAs. miR-26a is a functional miRNA that has merited previous investigation [29]. It is known that miR-26a plays a significant role in the growth, development and cell differentiation of different tissues [29]. Several studies have shown that miR-26a expression is disordered in a number of human tumors [19], [22], [24], [30], [31]. However, none of these studies is related to gastric cancer. In the current study, we used qRT-PCR and ISH to show that miR-26a levels in gastric cancer tissues were significantly lower than those in non-tumor tissues. Moreover, the miR-26a levels were associated with the clinical stage and presence of lymph node metastases. Kaplan-Meier survival analysis revealed that patients whose primary tumors displayed low expression of miR-26a had a shorter OS and RFS in GC. In addition, Cox proportional-hazards regression analysis showed that reduced miR-26a in tumors was a strong and independent predictor of shorter OS and RFS. Based on array data, it was previously reported that a combination of several miRNAs may be useful as prognostic markers in gastric cancer [32], [33]. Additionally, a single-miRNA, such as miR-218, can be a prognostic indicator [34]. However, these miRNAs have been investigated in only a few gastric cancer patients. Here, miR-26a may be useful as a prognostic marker to predict survival and relapse in gastric cancer.

This study showed that ectopic expression of miR-26a in GC cells impaired migration, invasion, proliferation and colony growth as well as induced apoptosis. Moreover, in vivo data demonstrated miR-26a inhibited tumor growth and metastasis. These in vitro and in vivo data further indicated that miR-26a functions as a tumor suppressor in gastric cancer. Several studies support our results. For example, this miRNA is decreased in NPC tissues and can attenuate cell growth and tumorigenesis by targeting EZH2 [22]. Hepatocellular carcinoma also exhibits reduced expression of miR-26a [19]. Systemic administration of this miRNA in a mouse model of HCC using an adeno-associated virus (AAV) results in inhibition of cancer cell proliferation, induction of tumor-specific apoptosis, and dramatic protection from disease progression without toxicity [35]. However, various recent studies revealed the oncogenic role of miR-26a in tumors such as glioma and cholangiocarcinoma. Huse et al. reported that miR-26a was overexpressed in high-grade glioma and directly targeted PTEN [23]. Similarly, miR-26a was found to be overexpressed in human cholangiocarcinoma tissues and to promote cholangiocarcinoma growth by activating B-catenin [24]. These controversial results suggested that the role of miR-26a was possibly tumor specific and highly dependent on its targets in different cancer cells. Various studies have shown that PTEN [23], EZH2 [21], [22], SMAD1 [36], CDK6 and cyclin E1 [37] are potential downstream target genes of miR-26a. In this study, FGF9, a new direct and functional target of miR-26a, was identified in GC. FGF9, also known as glial activating factor, is one of 23 members of the highly conserved FGF family. As a secreted, glycosylated 26-kDa protein, it has mitogenic effects on a variety of different cell types [26]. FGF9 has been shown to be implicated in different cancers such as ovarian endometrioid adenocarcinoma [26], hepatocellular carcinoma [27] and prostate carcinoma [28]. In our studies, we confirmed that FGF9 was a direct target of miR-26a in GC cells. To determine whether miR-26a suppresses GC growth and metastasis through repressing FGF9 expression, we found that FGF9 overexpression could rescue invasion and growth defects of miR-26a. These results suggested that miR-26a inhibits GC growth and metastasis partly by targeting FGF9.

Taken together, we observed downregulation of miR-26a in gastric cancer cells and demonstrated that miR-26a may act as an independent predictor of OS and RFS. We further found that miR-26a possesses the potency to suppress GC growth and metastasis by regulating FGF9. Our findings suggest miR-26a functions as a tumor suppressor in GC and holds promise as a prognostic biomarker and potential therapeutic target for GC.

## Materials and Methods

### Cell Culture

The gastric epithelial cell line GES-1 was purchased from the Beijing Institute for Cancer Research (Beijing, China). The GC cell lines MKN-28, SGC-7901, AGS, MGC-803, MKN-45, and BGC-823 were obtained from the Chinese Academy of Medical Science (Beijing, China). These cells were maintained at 37°C in an atmosphere of 5% CO_2_ in RPMI-1640 (MGC-803, BGC-823, MKN-28, SGC-7901), DMEM (GES-1) or F12 (AGS) medium supplemented with 10% fetal bovine serum, penicillin and streptomycin (Gibco BRL, NY, USA). All transfections used Lipofectamine 2000 ((Invitrogen, Carlsbad, USA)).

### Clinical Samples

All tissue samples used in the present study were collected from the Hunan Provincial Tumor Hospital (Changsha, Hunan, China). Written informed consent was obtained from all study participants. This study was approved by the Ethics Committee of Guangzhou Medical University Health Authority. The collection and use of tissues followed the procedures that are in accordance with the ethical standards as formulated in the Helsinki Declaration.

Tissue samples from 40 gastric cancer patients (23 male and 17 female; median age 59 years; range 40–84 years) were used for quantitative real-time PCR (qRT-PCR) analysis. Resected cancerous tissues (Tumor) and paired matched normal gastric tissues (Normal) were immediately cut, frozen in liquid nitrogen, and kept at −80°C until RNA extraction.

The tissue microarrays (TMAs), consisting of 126 GCs and 41 adjacent normal stomach tissues, were used for in situ hybridization analysis. The median age of gastric cancer patients at diagnosis was 57 years (range 31–82). The median follow-up time for overall survival (OS) and relapse-free survival (RFS) was 22 months and 15 months, respectively. All data of 126 GC samples, including age, sex, tumor site, histological grade, invasion depth (T stage), clinical stage, and lymph node metastasis were obtained from clinical and pathologic records and summarized in supplemental table 2.

### Quantitative RT-PCR Analysis

Total RNA was extracted from cells using TRIzol reagent (Invitrogen, Carlsbad, USA). Reverse transcription and qRT-PCR reactions were performed by using a qSYBR-green PCR kit (Qiagen, Germantown, USA) and U6 snRNA was used as an endogenous control. Fold change was determined as 2^−ddCt^. The Ct is the fractional cycle number at which the fluorescence of each sample passes the fixed threshold. The ddCt was calculated by subtracting the dCt of the reference sample (paired non-tumor tissue for surgical samples and GES-1 cell for six gastric cancer cell lines) from the dCt of each sample. The sequences of the specific primers for miR-26a and U6 snRNA were 5′-CTTCAAGTAATCCAGGATAGGC-3′ and 5′-ATTGGAACGATACAGAGAAGATT-3′, respectively.

### *In situ* Hybridization and Immunohistochemistry

Detection of miR-26a by in situ hybridization utilizing the DIG-labelled locked nucleic acid (LNA)-based probe specific for miR-26a (Exiqon, Vedbaek, Denmark) was performed according to the manufacturer’s instructions and U6 snRNA was used as an positive control. Brieﬂy, tissue microarray slides were deparaffinized, treated with proteinase K (5 min in 2 µg/ml protease K), washed in PBS and subsequently blocked endogenous peroxidase activity with 3% H_2_O_2_. Hybridization was performed at 52°C overnight after the addition of 50 nM of DIG-labelled LNA probes, followed by a stringency wash in SSC buffers. After blocking (2% sheep serum and 2 mg/ml BSA in PBS with Tween-20) at room temperature, the probe-target complex was visualized utilizing an anti-DIG-POD antibody, and DAB complex.

Immunohistochemistry was performed on tissue microarray sections using anti-FGF9 antibody (Santa Cruz, CA, USA). The complex was visualized with streptavidin/peroxidase and DAB complex, and nuclei counterstained with hematoxylin. In situ hybridization and immunohistochemistry results were scored by intensity (0–4) and the percentage of staining (0 to 100%). Relative expression was obtained by multiplying intensity by percentage. The slides were analyzed by two independent pathologists.

### Recombinant Vector

Recombinant lentiviruses containing miR-26a precursor or scramble sequences were purchased from SunBio (Shanghai, China). For luciferase analysis, the full-length FGF9 3′-UTR was amplified from human blood genomic DNA and then cloned into the downstream region of a firefly luciferase cassette in pMIR-REPORT luciferase vector (Ambion, Austin, TX, USA). The corresponding mutant constructs, in which the first six nucleotides complementary to the miR-26a seed-region were mutated by site-directed mutagenesis (Stratagene, La Jolla, CA, USA). To construct FGF9-expressing vector, FGF9 ORF cDNA was purchased from GeneCopeia (Rockville, MD, USA) and subcloned into the eukaryotic expression vector pcDNA3.1(+) (Invitrogen).

### Luciferase Reporter Assay

miR-26a or scramble lentiviruses and pMIR-3′UTR vector were co-transfected into HEK-293T cells. Renilla and firefly luciferase activities were measured with the Dual-Luciferase Reporter system (Promega, Madison, WI) 24 h after transfection. Firefly luciferase activity was normalized to Renilla luciferase expression for each sample. Each experiment was performed in triplicate.

### Western Blot

Western blotting was carried out as described previously [16]. Briefly, protein lysates were separated by 10% SDS-PAGE, and elec-trophoretically transferred to PVDF (polyvinylidene difluoride) membrane. Then, the membrane was incubated with mouse antibody against anti-FGF9 antibody (Santa Cruz, CA, USA) followed by HRP (horseradish peroxidase)-labeled goat-antimouse IgG (Santa Cruz, CA, USA) and detected by chemiluminescence. β-actin was used as a protein-loading control.

### Cell Proliferation Assay

Cells were plated on 12-well plates at the desired cell concentrations. Cell counts were estimated by trypsinizing the cells and performing analysis using a Coulter Counter (Beckman Coulter, Fullerton, USA) at the indicated time points in triplicate.

### Cell Migration and Invasion Assays

Cell migration was examined using wound-healing assays. An artificial “wound” was created on a confluent cell monolayer, and photographs were taken using an inverted microscope (Olympus, Tokyo, Japan) at 24 hours.

For cell invasion assay, cells were seeded onto the basement membrane matrix present in the insert of a 24-well culture plate (EC matrix, Chemicon, Temecula, CA) and fetal bovine serum was added to the lower chamber as a chemoattractant. After a further 48 hours, the non-invading cells and EC matrix were gently removed with a cotton swab. Invasive cells located on the lower side of the chamber were stained with Crystal Violet, counted and imaged.

### Colony Formation Assay

Six-well plates were covered with a layer of 0.6% agar in medium supplemented with 20% fetal bovine serum. Cells (1×10^4^) were prepared in 0.3% agar and seeded in triplicate. After the plates were incubated at 37°C for two weeks, the colonies were counted.

### Apoptosis Analysis

The apoptotic cells were evaluated by Annexin V-FITC and propidium iodine staining (BD, USA) and analyzed with a FACS Calibur instrument (BD, USA). The collected data were analyzed using FlowJosoftware.

### Mouse Xenograft Model

The gastric cancer model in nude mice was constructed as described before [25]. To evaluate the role of miR-26a in tumor formation, SGC-7901 cells infected with miR-26a or scramble viruses were propagated and inoculated subcutaneously into the dorsal flanks of nude mice (5 in each group). Tumor size was measured every 5 days. After 30 days, the mice were killed, necropsies were performed and the tumors were weighed. Tumor volumes were determined according to the following formula: A×B^2^/2, where A is the largest diameter and B is the diameter perpendicular to A. To assay miR-26a’s effect on tumor metastasis, SGC-7901 cells infected with miR-26a or scramble viruses were injected into the tail vein of nude mice (6 in each group). After 45 days, necropsies were performed. Numbers of micrometastases in lung per HE-stained section in individual mice were analyzed by morphology observation. The experimental protocols were approved by the Animal Research Protection Committee of Guangzhou Medical University. Standard animal care and laboratory guidelines were followed.

### Statistical Analysis

Comparisons between groups were analyzed by the *t-*test and x^2^ test. Overall survival curves and relapse-free curves were plotted using the Kaplan-Meier method, with the log-rank test applied for comparison. Survival was measured from the day of surgery. Variables with a value of *P*<0.05 according to the univariate analysis were used in subsequent multivariate analysis based on the Cox proportional hazards model. Spearman’s correlation tests were used to evaluate the pairwise expression correlation between miR-26a and FGF9 in GC tissues. All differences were statistically significant at the level of *P*≤0.05. Statistical analyses were performed using SPSS15.0 software.

## Supporting Information

Figure S1 **qRT-PCR measured the miR-26a expression levels in SGC-7901 and AGS cells infected with miR-26a lentivirus as well as GES-1 cells transfected with miR-26a inhibitors.**
(TIF)

Figure S2 **qRT-PCR measured the miR-26a expression levels of tumor tissues extracted from nude mice on the 30th day upon cancer cells injection.** All data are shown as mean±s.e.m.
(TIF)

Table S1 **Target genes that were predicted by all three algorithms (Targetscan, Pictar and Miranda).**
(XLS)

Table S2 **Characteristics of patients with gastric cancer.**
(DOC)
