# Supplementary figures and images for: miR-26a Suppresses Tumor Growth and Metastasis by Targeting FGF9 in Gastric Cancer
Source: PLoS One. 2013 Aug 28;8(8):e72662. doi: 10.1371/journal.pone.0072662 (PMC3756000; doi:10.1371/journal.pone.0072662)

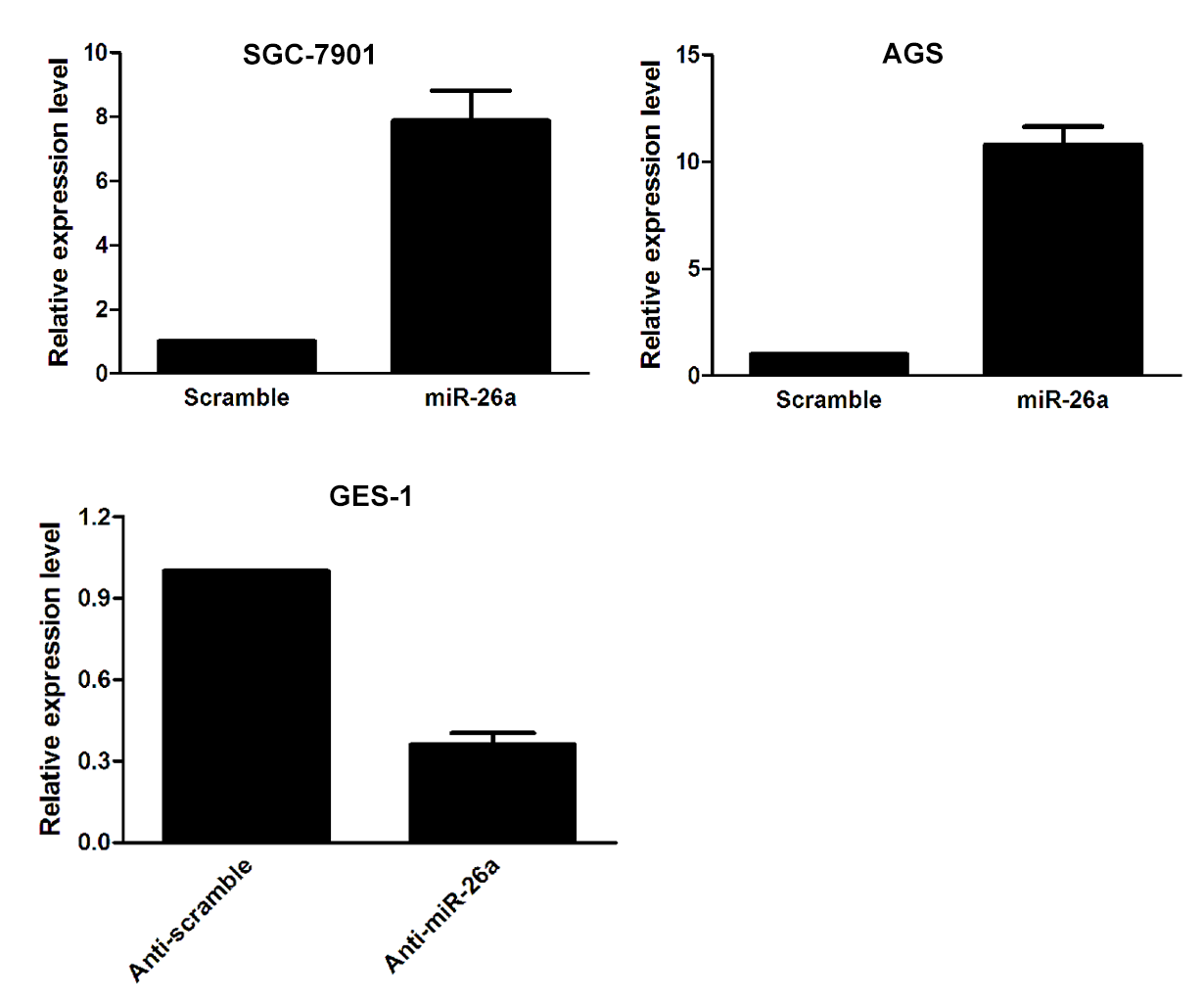

Supplement: Figure S1 — qRT-PCR measured the miR-26a expression levels in SGC-7901 and AGS cells infected with miR-26a lentivirus as well as GES-1 cells transfected with miR-26a inhibitors. (TIF) [file pone.0072662.s001.tif]

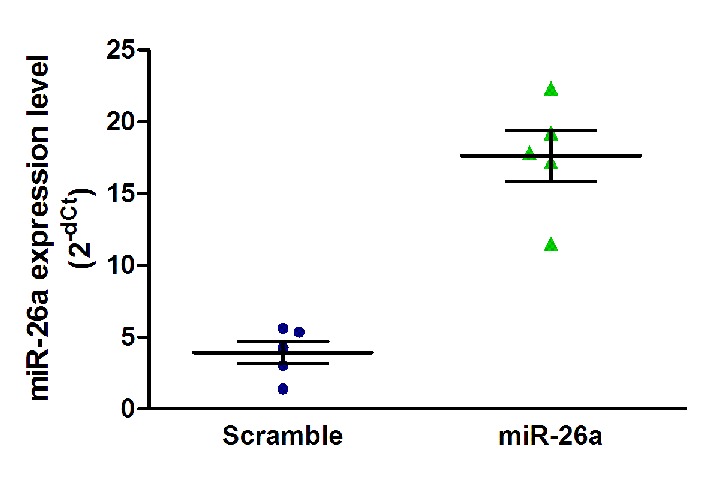

Supplement: Figure S2 — qRT-PCR measured the miR-26a expression levels of tumor tissues extracted from nude mice on the 30th day upon cancer cells injection. All data are shown as mean±s.e.m. (TIF) [file pone.0072662.s002.tif]
