# Supplementary material for: miR-26a Suppresses Tumor Growth and Metastasis by Targeting FGF9 in Gastric Cancer
Source: PLoS One. 2013 Aug 28;8(8):e72662. doi: 10.1371/journal.pone.0072662 (PMC3756000; doi:10.1371/journal.pone.0072662)
Supplement: Table S2 — Characteristics of patients with gastric cancer. (DOC) [file pone.0072662.s004.doc]

**Table S2.** Characteristics of patients with gastric cancer

| **Viable** | **Number of cases** |
| --- | --- |
| Age (years) |  |
| ＜60 | 73 |
| ≥60 | 53 |
| Gender |  |
| Male | 70 |
| Female | 56 |
| Tumor site |  |
| Cardiac | 20 |
| Body | 29 |
| Pylorus | 77 |
| Histological grade |  |
| Well | 9 |
| Moderate | 23 |
| Poor & Other | 94 |
| T stage |  |
| T1 | 12 |
| T2 | 59 |
| T3 | 44 |
| T4 | 11 |
| TNM stage |  |
| I | 8 |
| II | 43 |
| III | 59 |
| IV | 16 |
| Lymph node metastasis |  |
| Present | 88 |
| Absent | 38 |
